# Research “junkification” is caused by researchers, not journals

**DOI:** 10.3389/fpsyg.2026.1845947

**Published:** 2026-05-28

**Authors:** Simon S. Groome, Jose C. Yong, Norman P. Li

**Affiliations:** 1 School of Social Sciences, Singapore Management University, Singapore, Singapore; 2 School of Social and Health Sciences, James Cook University, Singapore, Singapore

**Keywords:** crowding out, evolutionary psychology, human capital, junkification of research, meta-science, research quality, scientific innovation, stabilizing selection

*Science seemed to move faster in those days…All papers were published in those days…You didn't have to go through the agony of doing three revisions for Cell…The standard was higher in 1953 than it is today—*James Watson, 2009

## 1 Introduction

Concerns about declining research quality have increasingly been framed as “junkification,” whereby academic output expands in volume while declining in epistemic value. A recent paper by Rhodes and Linnenluecke (2025) attributes this trend to changes in publishing, arguing that research quality erodes as journals and publishers become increasingly oriented toward output quantity and metrics, driven by monetization.

In 2009, Nobel Laureate James Watson remarked that scientific publishing in the mid-twentieth century was both easier and of a higher standard than today (Web of Stories, 2017). If publishing is much easier of late, as Rhodes and Linnenelucke (R&L) argued, then how could lower barriers to publication coexist with higher quality, as Watson claimed?

R&L suggest five mechanisms: the expansion of publishing outlets, including the proliferation of journals and special issues; platform incentives, whereby publishers respond to submission pressure by increasing output; metric-driven evaluation that rewards volume over substance; marketization or commodification, with publishers portrayed as extracting value from academic labor; and the underrepresentation of the Global South in research, suggesting that uneven global participation contributes to low-quality research.

While R&L describe important downstream consequences of junkification, they potentially misidentify the problem's origin. Drawing on evolutionary-economic theory, we contend that junkification is fundamentally a selection problem, arising from the expansion of publicly subsidized academic labor without commensurate demand-side constraints. We characterize this process as a shift in selection under conditions of institutional congestion. The subsequent so-called “commodification” and “monetization” of academic publishing is best understood as defensive responses to this upstream selection failure, not as its cause. We conclude by arguing that social science fields grounded in strong, integrative theoretical frameworks are comparatively resistant to junkification, precisely because they preserve high cognitive entry costs and limit low-value niche occupation, natural defenses against the overinflation of academic supply.

## 2 Human capital in academia

Over the past several decades, doctoral training and academic employment have expanded rapidly without commensurate demand (Cyranoski et al., 2011), while the criteria governing entry into research careers increasingly relies on subjective evaluations such as letters of recommendation, personal statements, and perceived “fit,” while subjective cognitive evaluations have fallen by the wayside (Landrum and Clark, 2005; Langin, 2022).

Particularly within psychology, this dilution was reinforced by changes in admissions practices. Surveys of graduate programs show a move away from objective cognitive indicators toward more subjective criteria. A large review of psychology graduate admissions reported that recommendation letters, personal statements, and undergraduate GPA ranked as the most important selection factors, while standardized tests were deemphasized (Landrum and Clark, 2005). More recently, the use of the GRE in psychology admissions has collapsed almost entirely (Langin, 2022).

This is an important shift because the GRE General Test is strongly g-loaded and predicts a wide range of outcomes relevant to academic success, including graduate GPA, faculty evaluations, productivity, citation impact, and placement in research-intensive institutions (Kuncel et al., 2004; Kuncel and Hezlett, 2007). These relationships persist after controlling for socioeconomic background, indicating that the GRE provides substantial incremental validity that does not reflect privilege (Sackett et al., 2009). Given that the GRE is strongly g-loaded and shows consistent associations with a range of graduate outcomes, whereas commonly prioritized subjective criteria such as letters of recommendation and personal statements exhibit only weak predictive relationships and limited incremental validity for graduate outcomes other than degree attainment (Kuncel et al., 2014; Murphy et al., 2009), the de-emphasis of the GRE alongside increased reliance on letters of recommendation and personal statements may move selection toward traits less directly tied to the generation of significant academic contributions.

The consequences of this change are consistent with the well-established relationship between intelligence and performance in complex work. Meta-analyses in industrial–organizational psychology show that cognitive ability measures are the strongest predictors of job performance (0.6 in high-complexity occupations; Hunter, 1980). Moreover, performance in cognitively demanding domains is highly non-linear: a minority of individuals account for a disproportionate share of output (Hunter et al., 1990). Even for medium-complexity work, extreme 1% performance groups differ by a factor of 12-to-1. Our contention is that lower standards combined with academic oversupply induce selection processes to move away from raw cognitive ability toward traits that facilitate survival in congested markets. Accordingly, modern academia may increasingly reward traits such as persistence, conformity, and tolerance for administrative burden, sometimes at the expense of traits associated with originality and intellectual risk-taking (Charlton, 2009).

With the prioritization of subjective means of selection, credential inflation follows naturally; formal qualifications become common, while their informational value about underlying ability diminishes (Cyranoski et al., 2011), leading to academic oversupply. This process is not unlike stabilizing selection. That is, in academia, conditions increasingly resemble a stabilizing biological selection regime that penalizes deviation from the mean and maintains a narrow range around an optimum (Endler, 1986). High-variance strategies, such as intellectual risk-taking and heterodox theorizing, are penalized, while low-variance, norm-conforming work is favored. This may be one reason why, in psychology, naturalistic experiments have been replaced by a myriad of cheap online surveys (Baumeister et al., 2007).

R&L are right that contemporary science relies heavily on quantitative signals (e.g., journal rank, citation counts), but they mistake a symptom for a cause. Once output expands, status signals dilute such that even excellent work becomes harder to detect against a background of competent but redundant work. Selection predictably moves from direct, cognitively feasible, and heuristic quality evaluation (arguably more accurate; Gigerenzer and Gaissmaier, 2011) toward quantitative metrics. This is the point at which conformism and prestige bias predominates instead of direct evaluation (Boyd and Richerson, 1985), causing unoriginality and bandwagoning (see Section 6). R&L are correct that leaning on quantitative proxies imperfectly correlates with truth or importance, but it's a necessary evil at this point.

This creates a second, reinforcing dynamic, namely recognition bottlenecks. Scientific prestige, like prestige in most domains of life, is partly winner–take–all and constrained by finite attention (Frank and Cook, 1996). When grant panels must sort hundreds of CVs or submissions, easily understandable, low-variance norm-conforming and incremental work is a safer bet than high-variance originality, even if the latter has greater upside potential. This is possibly where today's “publish or perish” culture comes from, and is why, arguably, some of the most eminent scientists (e.g., Darwin, whose work was very slow to be accepted) may not succeed in academia today (Simonton, 1999). From a Darwinian perspective, since scientific progress depends disproportionately on the extreme right tail of creativity and ability, restricting variance is enough to reduce breakthroughs without any obvious collapse in mean competence (Simonton, 1999).

## 3 Journals and publishers

Given the selection failure produced by subsidized oversupply, journals face a predictable coordination problem. Historically, journals did not need extensive procedural barriers because contributor pools were already highly selected (Web of Stories, 2017). However, as the academic labor market expanded, this equilibrium broke down. Submission volumes rose sharply, driven not by a sudden increase in breakthrough ideas (as noted by R&L) but by the overwhelming growth of the academic workforce itself, as well as the advent of technologies that make research more efficient. In response to the glut of manuscripts being produced, journals proliferated and output increased. Crucially, this expansion of journals did not create low-quality research; it absorbed it.

Claims that this process reflects excessive “marketization,” “commodification,” and “neoliberal logics” may further mischaracterize the problem. The oversupply of academics, and thus the demand for publication by those same suppliers (a large source of demand for papers comes from the authors themselves, who are also the suppliers!), means that authors rarely bear the true opportunity costs of publication, and face no meaningful accountability for marginal, low-grade research. Article processing charges in open-access models simply function as absorbers of excess supply. In other words, they are transfer mechanisms that recycle public funds from universities and funders to publishers. This is a process mediated by status-seeking academics who need to publish more papers to effectively compete, not by profit-seeking journals *per se*. In such a distorted market, boilerplate claims about value extraction distract from the real problem, which stems from oversupply in the first place.

## 4 Academic crowding out

From an economic perspective, the consequences of academic (and thus, journal) oversupply constitutes a type of “crowding out,” whereby debt-funded fiscal policy displaces private activity by monopolizing scarce resources or by distorting incentives in ways that reduce the viability of private, higher quality alternatives (Blanchard, 2008).

To extend the analogy to junkification, when entry into a profession is funded largely through public expenditure and insulated from price signals (or, in this case, reputational scarcity), the volume of labor can grow far beyond what downstream consumers of output can absorb.

On the other hand, where submission pressure is low and contributor quality is high, pay-to-publish models cannot survive. The causal direction therefore runs from oversupply to journal behavior, not the reverse. That some publishers profit from this arrangement is neither surprising nor diagnostic, but a feature of a system in which demand for publication (by authors, not readers) is guaranteed to be higher than what traditional, high-quality journals can supply.

## 5 Global south exclusion

The junkification thesis further attributes declining research quality to inequality and exclusion, suggesting that the under-representation of researchers and samples from countries in the Global South degrades scientific knowledge. Yet, no causal mechanism is specified by which exclusion reduces research quality, nor is there any evidence cited that such exclusion is taking place at all.

From the Retraction Watch database, the top retraction rates (per 10k papers) were in Saudi Arabia (30.6), Pakistan (28.1), Russia (24.9), China (23.5), Egypt (18.8), Malaysia (17.2), Iran (16.7), and India (15.2), dominated by Middle Eastern, African, and South Asian countries (Van Noorden, 2023; Ioannidis et al., 2025). It is important to note that cross-national variation in retraction rates is unlikely to reflect a single underlying cause, as differences in infrastructure, funding environments, training systems, and publication incentives may all shape the conditions under which research is produced and evaluated. Nevertheless, the majority of these large-scale retraction waves have been driven by fabricated peer review, falsified data, and industrial-scale paper mills. Publishers have issued mass retraction notices citing a systematic manipulation of the publication process, often involving dozens or hundreds of papers at a time (Li et al., 2024; Li and Shen, 2024). This is junkification *par* excellence. Hence, the problem appears not to be exclusion, but rather blind inclusion without consistent selection and quality control mechanisms. Appeals to concepts such as “coloniality of knowledge” reframe a selection-and-incentives problem as a moral narrative about representation without evidence, while ignoring the most visible empirical metric of low research integrity. A related manifestation of inclusion-without-selection can be seen in the anti-WEIRD phenomenon, a type of “empiricist cataloging” that prioritizes data accumulation over theory testing (Kanazawa, 2024). That is, the accumulation of descriptive differences across populations does little to test important theories or advance understanding. Rather than refining hypotheses, post-WEIRD bandwagoning has incentivized the occupation of increasingly narrow empirical niches in the literature. This kind of research bandwagoning is a good example of junkification, as more papers are generated to report incremental data rather than offer impactful insights and theoretical advances.

## 6 Why evolutionary psychology is comparatively resistant to junkification

If junkification arises from weak selection and unconstrained niche occupation (whereby almost any phenomenon can be rendered publishable through novel framing), then we can speculate the fields that impose strong theoretical constraints may be relatively resistant. Evolutionary psychology provides a clear example. Many areas of psychology are organized primarily around the domain of interest. Within social psychology, for instance, lies numerous unconnected explanations for various phenomena (Kenrick et al., 2010). By contrast, evolutionary psychology constrains explanation through a common theoretical framework (Buss, 1995; Koç, 2025; Yong, 2025), which limits the extent to which arbitrary combinations of concepts and keywords can generate publishable findings. Hypotheses must be linked to plausible selection pressures, fitness-relevant outcomes, and mechanistic pathways shaped by natural or sexual selection.

This, of course, does not imply complete consensus. Philosophical and methodological debates within evolutionary psychology are well documented (Egeland, 2024; Laland and Brown, 2002), and the field is not immune to politically-based biases. But, even amid disagreement, participants share a requirement that explanations advance our understanding of central theoretical tenets. This requirement imposes high barriers to entry and sharply limits low-value niche occupation.

## 7 Conclusion

To avoid junkification, reforms can focus on the incentives governing the production of research rather than the behavior of journals alone. While many of the claims made here are only theoretical, future work can examine whether variation in labor supply and evaluation meaningfully affect research novelty. First, universities can restore stronger selection into research careers, thereby resisting the credential inflation that dilutes signals of talent, including reweighting admissions toward standardized testing. Second, more weight can be placed on papers of higher quality than large portfolios of incremental work. Finally, strengthening theoretical integration across fields (akin to evolutionary psychology) can help constrain the proliferation of low-value empirical niches.
